# Why do we feel close to a person who expresses gratitude? Exploring mediating roles of perceived warmth, conscientiousness, and agreeableness

**DOI:** 10.1002/pchj.682

**Published:** 2023-09-20

**Authors:** Tatsuya Imai

**Affiliations:** ^1^ Department of British and American Studies Nanzan University Nagoya Japan

**Keywords:** agreeableness, apology, closeness, conscientiousness, gratitude, warmth

## Abstract

The literature suggests that expressed gratitude improves the interpersonal relationship between a beneficiary and a benefactor. However, there is little research that has explored why thanking provides these positive effects, so this study investigated thanking mechanisms to explain reasons why people feel close to a beneficiary who expresses gratitude. This study also examines the effects of apologies, which are sometimes used to show gratitude in Japan. In this experimental study, 671 Japanese participants reported their perceived closeness, warmth, conscientiousness, and agreeableness to a hypothetical beneficiary who expressed gratitude, apologies, or both after a benefit was provided. The results revealed that benefactors who received a message indicating gratitude and both gratitude and apologies reported higher levels of closeness toward a beneficiary than those who received a message with only apologies and a message without either gratitude or apologies. A structural equation model further indicated that warmth and conscientiousness mediated the link between expressed gratitude/apologies and perceived closeness.

The literature indicates that expressed gratitude enhances an interpersonal relationship between a benefactor and a beneficiary. For example, benefactors who receive gratitude are motivated to affiliate with (Williams & Bartlett, 2015), disclose themselves to (Imai, 2022), and provide help to (Grant & Gino, 2010) a beneficiary. The research, however, provides limited knowledge on why expressed gratitude functions to develop a relationship between a beneficiary and a benefactor. This study attempted to examine the mechanism by which expressed gratitude helps improve an interpersonal relationship. This study also investigated if and how expressed *apologies* could function as a facilitating factor to improve an interpersonal relationship in the same way as gratitude does. In Japan, apologies are sometimes used to show gratitude when a benefit is provided (e.g., Kotani, 2002; Long, 2010), suggesting the possibility that expressed apologies, like expressed gratitude, enhance the relationship between a beneficiary and a benefactor.

One of the main focuses of previous studies on expressed gratitude was investigating the mechanism through which expressed gratitude leads to positive relationship outcomes (Grant & Gino, 2010; Williams & Bartlett, 2015). However, the research has focused on a limited number of mediators, such as self‐efficacy and social worth (Grant & Gino, 2010).The current study proposes other candidates as mediators, such as warmth, conscientiousness, and agreeableness, based on theoretical accounts such as self‐enhancement theory (Alicke & Sedikides, 2009) and interdependence theory (Thibaut & Kelley, 1959). The examination of new possible mediators could result in a theoretical contribution to the field of research examining how expressed gratitude helps facilitate interpersonal relationships (e.g., Algoe, 2012). Further, while previous studies on expressed gratitude have been conducted mainly in Western countries such as Australia (Williams & Bartlett, 2015) and the United States (Grant & Gino, 2010), the current study was conducted in Japan, where people often use apologies to express gratitude (Kotani, 2002; Long, 2010). This results in the question of, when receiving a gift from a Japanese person, should one say, “thank you,” “sorry,” or both? In light of the above, this study tried to answer the question with an attempt to investigate the mechanism of how expressed gratitude and apologies bind people together.

## Influences of expressed gratitude and apologies

Gratitude is a positively valenced emotion that can be evoked when a benefactor provides a positive outcome to someone (Algoe et al., 2016). Gratitude is typically conveyed to a benefactor by thanking, which gives the benefactor credit for the positive outcome (Chaudhry & Loewenstein, 2019). The existing literature indicates that expressed gratitude improves the quality of interpersonal relationships in various contexts. Participants who expressed gratitude to a friend reported higher levels of the communal strength of the friendship compared with those who only had grateful thoughts about the friend (Lambert et al., 2010). Expressed gratitude in marital couples was found to be an important factor contributing to marital satisfaction (Schramm et al., 2005). Some research focused on the impact of expressed gratitude on a *benefactor*'s perceptions. Algoe et al. (2013) revealed that participants who heard gratitude expressed by their romantic partner forecasted a positive change in their relationship satisfaction over 6 months. Further, Algoe et al. (2016) found that the degree to which a romantic partner expressed gratitude was linked to their partner's perception of the expressor's responsiveness. Benefactors in a study conducted by Imai (2022) were motivated to disclose themselves to a beneficiary after they received gratitude from the beneficiary.

One of the goals of this study is to extend the above literature by testing the positive effects of expressed gratitude in Japan, where apologies are sometimes used to show gratitude (Kotani, 2002; Long, 2010). Japan is a country where people are commonly concerned about indebtedness to others (Benedict, 1946; Naito & Sakata, 2010), so when a benefit is provided, it is not uncommon for Japanese beneficiaries to try to minimize the impact of the indebtedness to a benefactor by apologizing (Coulmas, 1981). While thanking is a way to give a benefactor credit for a positive outcome, apologizing is a way for a beneficiary to take blame for a negative outcome, such as the indebtedness to the benefactor (Chaudhry & Loewenstein, 2019). For example, in a favor‐asking situation, Japanese participants perceived that apologies could moderate the indebtedness to someone who received a request (Lee et al., 2012). It is also natural for Japanese to use both gratitude and apologies at the same time, to let a benefactor know that they appreciate the benefactor's support and they recognize that they inconvenienced the benefactor (Yamamoto, 2003). In fact, a study by Imai (2022) found some positive effects of received gratitude and apologies on Japanese benefactors' perceptions, such as elevated social worth and more intention when disclosing themselves to a beneficiary. Considering both of these factors, it is critical to test if expressed gratitude enhances interpersonal relationships even in Japan, where apologies can be used to show gratitude.

The current study specifically focused on how perceived *closeness* toward a beneficiary would be impacted by different messages. Closeness can be defined as an individual's sense of being interconnected with another (Gächter et al., 2015). Closeness should be examined as a factor that expressed gratitude could impact because closeness is the key ingredient within various relationships, such as friendship (Parks & Floyd, 1996), romantic relationships (Adams et al., 2001), and family (Mikulincer et al., 1993), where gratitude is commonly expressed. Specifically, closer friends are more likely to trust and provide social support for each other than less close friends (Salazar, 2015). The higher the closeness that partners experience in a romantic relationship, the less likely the relationship is to dissolve (Le et al., 2010). Perceived closeness within a marital couple impacts how a person in the relationship psychologically adapts to his or her illness (Manne & Badr, 2008). Closeness within a family is positively correlated with children's school adjustment (Lee et al., 2007). This study investigated the link between received gratitude/apologies and closeness, which is the key to maintaining various types of interpersonal relationships.

Benefactors (Japanese participants) who receive a message expressing gratitude from a beneficiary feel closer to the beneficiary than do those who receive a message without gratitude and apologies.

Benefactors (Japanese participants) who receive a message expressing apologies from a beneficiary feel closer to the beneficiary than do those who receive a message without gratitude and apologies.

Benefactors (Japanese participants) who receive a message expressing both gratitude and apologies from a beneficiary feel closer to the beneficiary than do those who receive a message without either of them.

## Mechanisms of the positive effects of expressed gratitude on closeness

Why are people who thank others likely to be desirable relationship partners? Several theories could explain the link between receiving expressed gratitude and closeness to the expressor: self‐enhancement theory, self‐expansion theory, and interdependence theory. According to these theories, we tend to feel close to those who thanked us because the thankers could help us enhance our self‐view, expand our identity, and give us positive outcomes. Based on self‐enhancement theory, people think and behave in a way that maintains or advances their desired self‐views (Alicke & Sedikides, 2009). Consistent with the theoretical idea, people in a romantic relationship with a partner who evaluates them positively feel closer to the partner (Swann Jr et al., 1994). Based on these explanations, it is plausible that people feel close to others who express gratitude because receiving gratitude enhances their self‐views through the perception that they have had a positive impact on the expressor's life (Grant & Gino, 2010). Another theory that could explain the reason for the increases in closeness that gratitude receivers experience toward gratitude expressors is self‐expansion theory. The core idea of the theory is that people are motivated to expand their identity through entering into relationships (Aron & Aron, 1986; Aron & Aron, 1996). Based on this theory, people seek new experiences by becoming close to others who could expand their sense of self. Integrating this idea into the situation where benefactors receive gratitude from a beneficiary, they could experience an expansion of their identity due to their belief that they could make a positive change in the world, and that may lead to the increase in closeness toward the gratitude expressor.

Past studies often depended on the third theoretical perspective, which is interdependence theory, to examine the reason why people tend to feel close to an individual who thanked them. The main idea of Thibaut and Kelley (1959) in interdependence theory is that people predict the outcome of the relationship they are in, and this outcome, which is called the *relational outcome*, could influence how they perceive and evaluate the relationship. The outcome is calculated based on the *rewards* (e.g., desirable and pleasant experiences*)* and *costs* (e.g., undesirable and negative experiences) that the relationship brings. In order for a relationship to be close, high levels of relational outcome (rewards – costs) are necessary (Guerrero et al., 2017; Miller, 2017). The reason for the increase in closeness that benefactors experience toward a beneficiary expressing gratitude might be due to the predicted relational value that the benefactor perceives within the relationship. For example, Imai (2022) revealed that benefactors receiving gratitude from a beneficiary were motivated to disclose themselves to the beneficiary partly because of their prediction of high values attached to the relationship with the beneficiary. The research indicated that people feel close to those who thanked them because the relationship with the thanker is considered to be beneficial owing to the prediction that the thanker could bring positive outcomes. Based on interdependence theory, the current study tried to extend the literature by exploring other possible mechanisms to explain the link between expressed gratitude/apologies and enhanced closeness, focusing on the following possible mediators: perceived warmth, conscientiousness, and agreeableness.

According to previous studies (Ames & Bianchi, 2008; Klimstra et al., 2013), agreeableness tends to be strongly associated with conscientiousness and warmth, but the literature also suggests that the three factors are distinct concepts. First, agreeableness and conscientiousness are two of the five factors of personality according to the five‐factor model (Digman, 1990). The five factors are neuroticism, extraversion, openness, agreeableness, and conscientiousness, and the five factors are theoretically distinct from each other (Digman, 1990). Second, John and Srivastava (1999) argued that it theoretically made the most sense to consider warmth a *facet* of agreeableness.

*Warmth*. It is critical to display warmth to form a desirable relationship with others (Fiske et al., 2007). Perceived warmth is also one of the most important characteristics when judging others to be a desirable friend, romantic partner, or spouse (Sprecher & Regan, 2002). The impression of being seen as warm could be achieved through thanking and apologizing. The literature suggests that thanking could help thankers look warm (Chaudhry & Loewenstein, 2019; Williams & Bartlett, 2015). In fact, Williams and Bartlett (2015) found that perceived warmth mediated the association between expressed gratitude and a benefactor's intentions to affiliate with a beneficiary, although they did not assess perceived closeness as an outcome. Wei and Ran (2019) also indicated that apologizing made apologizers look warm because apologizing showed that apologizers were willing to take the blame for negative outcomes without blaming others. Taken together, these previous studies contributed to the formulation of the hypothesis in the current study that perceived warmth could function as a mediator explaining the link between expressed gratitude/apologies and a benefactor's perceived closeness to a beneficiary.

*Conscientiousness*. Conscientiousness is a personality trait indicating that an individual has self‐discipline and follows social norms regarding impulse control (John & Srivastava, 1999; Roberts et al., 2009). Conscientious people have advantages in forming desirable interpersonal relationships because others are likely to trust them (Hill et al., 2014), so being perceived to be conscientious is key to the development of personal relationships. If beneficiaries express gratitude, they could make a benefactor perceive how conscientious they are because they seem to follow the social rule that gratitude should be expressed when receiving a benefit (Eibach et al., 2015). Apologies may also help apologizers appear conscientious because they could show that apologizers sincerely admit to wrong‐doing (Chaudhry & Loewenstein, 2019), suggesting their trustworthiness, which is a critical component of conscientiousness (Evans & Revelle, 2008). Therefore, perceived conscientiousness could function to mediate the link between expressed gratitude/apologies and the degree to which a benefactor feels close to a beneficiary.

*Agreeableness*. Agreeableness is defined as the propensity to be considerate, cooperative, sympathetic, and kind (Thompson, 2008). Agreeableness has been found to be one of the most important characteristics when selecting someone as an ideal relational partner (Jensen‐Campbell et al., 2010; Regan, 1998; Wortman & Wood, 2011). The existing literature suggests that grateful and thankful people tend to consider themselves to be agreeable (McCullough & Tsang, 2004), so expressing gratitude may help the expressor look agreeable. Also, a previous study revealed that a conciliatory gesture through apologies made the transgressor appear agreeable (Tabak et al., 2012). Taking into consideration the theoretical accounts above, perceived agreeableness may work as a mediator linking the association between expressed gratitude/apologies and a benefactor's closeness toward a beneficiary.

A message showing gratitude, apologies, or both gratitude and apologies is indirectly associated with a benefactor's perceived closeness toward a beneficiary through the benefactor's perception of warmth, conscientiousness, and agreeableness toward the beneficiary.

## METHOD

### Participants and procedures

A total of 671 Japanese university students (*M*_*age*_ = 21.60 years, *SD* = 1.61) were recruited for this study through two methods: 587 students participated in this study through a service run by a sample recruitment company, and 84 participated in this research through snowball sampling. The total sample size is considered sufficient based on the results of the G*Power analysis (multivariate analysis of variance [MANOVA]; four groups, four dependeng variables [DVs], *α* = .05, power = .8, *f* ^*2*^ independent variable [IV] = .15 based on Cohen, 1988) showing that a total of 85 participants are necessary for the main analyses in this study (F. Faul et al., 2007; F. Faul et al., 2009). A total of 137 participants reported that they were male, and 520 reported that they were female. Nine participants reported that their gender was categorized into *other*, and five participants preferred to not report their gender. The different methods of recruitment and gender (but not age) were found to be correlated with some of the dependent variables, so the effects of these factors were controlled for when analyzing the data. This study obtained Institutional Review Board approval from the Research Ethics Committee of Nanzan University. The data utilized in this study were part of a larger project, so other variables besides the ones collected for this study were also collected.

After answering questions assessing their demographic information, participants read a message that was purported to have been sent by a friend at the same university. In the message, they were asked by the friend to read a statement of purpose that the friend had written to apply to a study‐abroad program. After that, they were instructed to write feedback on that statement. After providing the feedback, they automatically received a reply message from the friend, the content of which randomly differed according to four different conditions: control (*n* = 175), gratitude (*n* = 160), apologies (*n* = 169), and both (*n* = 167).

The message in the *control* condition read “I read you advice! ( ) Actually, besides ABC University, I need to submit another statement of purpose for XYZ University. Can you read and give me some advice on the document, too? If you are too busy, that's OK. Your help would be appreciated!” In the *gratitude* condition, the following statement was inserted in the parentheses: “Thank you so much! I appreciate it!” In the *apology* condition, the following statement was inserted in the parentheses: “I'm sorry to bother you when you are busy!” Finally, in the *both* condition, the following statement was inserted in the parentheses: “I'm sorry to bother you when you are busy! Thank you so much! I really appreciate it!” All messages were written in Japanese. After reading the reply, the participants were asked to report their perceptions toward the hypothetical friend. According to their responses to three items assessing the realism (“The situation described in the scenario could occur for real,” “The situation described in the scenario was realistic,” and “I could face the situation described in the scenario”), the participants felt that this scenario was reasonably realistic (*M* = 3.54 out of 5; a higher score indicates higher realism), without significant differences among conditions (*F* = 0.94, *p* = .421, η^2^ = .004, 95% confidence intervals [95% CIs] [0, .015]).

### Measures

After reading the reply from the hypothetical friend, the participants responded to items related to the following measures: closeness, warmth, conscientiousness, and agreeableness. Table 1 indicates these variables' reliabilities (Cronbach's αs), means, and standard deviations. Measures originally written in English were back‐translated into Japanese by the researcher and another person, both of who use English and Japanese fluently.

**TABLE 1:** Reliabilities, means, standard deviations, and correlations (*n* = 671).

	1	2	3	4	α	*M*	*SD*
1. Closeness	‐				‐	3.25	1.36
2. Warmth	.41**	‐			.83	3.43	1.10
3. Conscientiousness	.34**	.54**	‐		.87	3.16	1.06
4. Agreeableness	.33**	.66**	.63**		.72	3.60	0.79

* *p* < .05;

** *p* < .01.

*Closeness*. The participants' perceived closeness toward the hypothetical friend was measured by the “Inclusion of the Other in the Self” (IOS) scale, developed by Aron et al. (1992). This scale asked participants to assess their relationship with the hypothetical friend by selecting one out of seven pairs of increasingly overlapping circles that ranged from barely touching to almost completely overlapping. They selected the pair of circles that best described their relationship with the friend. The score ranged from 1 to 7, with higher scores indicating higher levels of closeness (1 = *no overlap*; 2 = *little overlap*; 3 = *some overlap*; 4 = *equal overlap*; 5 = *strong overlap*; 6 = *very strong overlap*; 7 = *most overlap*). The use of this scale is considered appropriate because, compared with other closeness scales such as the Relationship Closeness Inventory (Berscheid et al., 1989) focusing on the situation for North American college students, the IOS scale is so intuitive and simple that people from non‐Western countries can use it without trouble (Gächter et al., 2015). Because of its usefulness, it has been successfully used in various countries, including China and Japan (Karremans et al., 2011).

*Warmth*. The participants' perceived warmth toward the hypothetical friend was assessed by the 5‐item scale of interpersonal warmth developed by Williams and Bartlett (2014). Example items are “The friend is friendly” and “This friend is likable.” A 5‐point Likert scale was used (1 = *strongly disagree*, 5 = *strongly agree*), with higher scores indicating more warmth. The Cronbach's *α* value was .83.

*Conscientiousness*. The participants' perceived conscientiousness toward the hypothetical friend was examined through 7 items, which particularly assessed conscientiousness, from a shortened form of the Big‐Five Scale developed by Namikawa et al. (2012). Example items are “This friend is careless” and “This friend is lazy.” Some items were reverse‐coded. A 5‐point Likert scale was used (1 = *strongly disagree*, 5 = *strongly agree*), with higher scores indicating more conscientiousness. The Cronbach's *α* value was .87.

*Agreeableness*. Six items from a shortened form of the Big‐Five Scale developed by Namikawa et al. (2012) were used to assess the participants' perceived agreeableness toward the hypothetical friend. Example items are “This friend is generous” and “This friend is selfish.” Some items were reverse‐coded. A 5‐point Likert scale was used (1 = *strongly disagree*, 5 = *strongly agree*), with higher scores indicating more agreeableness The Cronbach's *α* value was .72.

## RESULTS

The following results indicated that the manipulation worked successfully. Gratefulness in the message was measured by the following two items: “This message included the friend's grateful feeling toward you” and “This message included expressions showing ‘thank you.’” Apologies in the message were measured by the following two items: “This message included the friend's apologetic feeling toward you” and “This message included expressions showing ‘I am sorry.’” For both measures, a 5‐point Likert scale was used (1 = *strongly disagree*, 5 = *strongly agree*). Participants in the *gratitude* condition (*M* = 3.98, *SD* = 0.81) and the *both* condition (*M* = 4.00, *SD* = 0.83) reported that the hypothetical friend showed more appreciation than did participants in the *apology* condition (*M* = 3.06, *SD* = 0.93) and those in the *control* condition (*M* = 2.19, *SD* = 0.94) *F* (3, 667) = 162.93, *p* < .001, η^2^ = .423, 95% CIs [.367, .468]. Further, participants in the *apology* condition (*M* = 3.48, *SD* = 0.96) and the *both* condition (*M* = 3.28, *SD* = 1.08) reported that the hypothetical friend expressed more apologies than did participants in the *gratitude* condition (*M* = 2.67, *SD* = 1.03) and those in the *control* condition (*M* = 2.38, *SD* = 1.03), *F* (3, 667) = 45.97, *p* < .001, η^2^ = .171. All the analyses described below were conducted while controlling for the effects of the different recruiting methods and gender, because these factors were correlated with some of the dependent variables.

To test H1a‐c predicting that participants in the *gratitude*, *apology*, and *both* conditions would feel closer to the hypothetical friend than those in the *control* condition (i.e., those who received a message without either gratitude or apology), a one‐way MANOVA was used to examine the influence of different messages (i.e., *control*, *gratitude*, *apology*, and *both*) on closeness, warmth, conscientiousness, and agreeableness. The mediators were also included in the model for supplementary analyses. The results generated a significant influence of various messages, Wilks' lambda = .881, *F* (9, 1613) = 9.57, *p* < .001, η^2^ = .041, so analyses of variance (ANOVAs) were further performed.

As shown in Table 2, a one‐way ANOVA revealed that participants in the *gratitude* and *both* conditions reported that they felt closer to the hypothetical friend than did participants in the *control* condition (*F* [3, 665] = 8.44, *p* < .001, η^2^ = .037). The score of closeness in the *apology* condition did not differ from those in any other conditions. The results are consistent with H1a and H1c, but not with H1b.

**TABLE 2:** Effects for conditions using the Bonferroni correction.

Dependent variables	Control	Gratitude	Apologies	Both
	(*n* = 175)	(*n* = 160)	(*n* = 169)	(*n* = 167)
Closeness	2.85^a^	3.47^b^	3.21^ab^	3.49^b^
	(1.23)	(1.26)	(1.40)	(1.44)
Warmth	2.89^a^	3.69^c^	3.37^b^	3.80^c^
	(1.01)	(1.02)	(1.06)	(1.08)
Conscientiousness	2.80^a^	3.40^b^	3.05^a^	3.42^b^
	(1.09)	(0.95)	(1.01)	(1.06)
Agreeableness	3.30^a^	3.76^bc^	3.54^b^	3.83^c^
	(0.79)	(0.72)	(0.76)	(0.81)

*Note*: Standard deviations are reported in parentheses. Different superscripts indicate significant differences based on post hoc analyses using the Bonferroni correction test at *p* < .05.

As summarized in Table 2, the following results for supplementary analyses indicated that different messages had an impact on participants' perceptions of warmth, conscientiousness, and agreeableness, which are hypothesized to work as mediators. A one‐way ANOVA revealed that participants in the *gratitude*, *apology*, and *both* conditions reported that the hypothetical friend was warmer than did participants in the *control* condition (*F* [3, 667] = 26.06, *p* < .001, η^2^ = .105). Also, the scores of perceived warmth in the *gratitude* and *both* conditions were higher than the score in the *apology* condition.

Further, a one‐way ANOVA indicated that participants in the *gratitude* and *both* conditions reported that the hypothetical friend was more conscientious than did participants in the *apology* and *control* conditions (*F* [3, 666] = 13.94, *p* < .001, η^2^ = .059).

The results also indicated that participants in the *gratitude*, *apology*, and *both* conditions reported that the hypothetical friend was more agreeable than did participants in the *control* condition (*F* [3, 666] = 16.52, *p* < .001, η^2^ = .069). Also, the score of agreeableness in the *both* condition was higher than that in the *apology* condition. That is, perceived closeness, warmth, conscientiousness, and agreeableness were found to be influenced by different messages.

**FIGURE 1:**
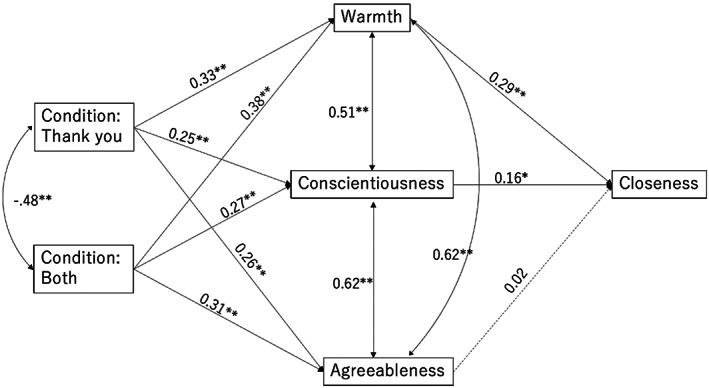
Results of the structural equation model. Solid lines represent significant paths. Dashed lines represent nonsignificant paths. Numbers indicate standardized beta coefficients. *p < .01, **p < .001.

To examine the second hypothesis, predicting that perceived warmth, conscientiousness, and agreeableness toward the hypothetical friend would mediate the link between different messages and closeness, structural equation modeling (SEM) was conducted using Mplus (Figure 1). As the results for H1 indicated above, the scores of closeness in the *gratitude* and *both* conditions were significantly higher than that in the *control* condition, but not than that in the *apology* condition. Baron and Kenny (1986) argued that there should be a statistically significant link between a predictor and an outcome in order to perform the mediation. Because the message showing apologies did not have a significant impact on closeness, the participants in the *apology* group were excluded in the analyses of SEM, and consequently the number of remaining participants was 502. Correlations among variables used for SEM are shown in Table 3.

**TABLE 3:** Correlations among variables used for structural equation modeling (*n* = 502).

	1	2	3	4	5	6	*M*	*SD*
1. Condition: Thank you	‐						‐	‐
2. Condition: Both	−.48**	‐					‐	‐
3. Closeness	.11*	.12**	‐				3.26	1.34
4. Warmth	.15**	.23**	.40**	‐			3.45	1.11
5. Conscientiousness	.13**	.15**	.33**	.56**	‐		3.20	1.08
6. Agreeableness	.12**	.18**	.32**	.66**	.66**	‐	3.62	0.81

*Note*: Conditions were dummy‐coded.

* *p* < .05;

** *p* < .01.

The model shown in Figure 1, including message types as a predictor, warmth, conscientiousness, and agreeableness as mediators, and closeness as an outcome, revealed a good fit: χ^2^(10) = 9.04, *p* = .53, CFI = 1.00, RMSEA = 0.00 (90% CIs [.00, .05]), SRMR = .01 based on the following criteria: CFI > .95, RMSEA < .06, SRMR < .08 for a good model fit (Hu & Bentler, 1999). Indirect effect analyses were conducted through the 5000 bootstrapping procedure, and the results showed four significant indirect paths. If the direct paths between predictors (i.e., conditions of different messages) and an outcome (i.e., closeness) were added in the model, the effects of these paths were not significant, indicating a possibility that the mediators fully mediated the links between predictors and an outcome.

With regard to the first significant indirect path, a message with gratitude was linked to closeness through warmth (*β* = .10, *p* < .001, 95% CIs [.045, .145]) (i.e., expressed gratitude → warmth → closeness). Second, a message with gratitude was linked to closeness through conscientiousness (*β* = .04, *p* = .01, 95% CIs [.010, .071]) (i.e., expressed gratitude → conscientiousness → closeness). Third, a message with gratitude and apologies was linked to closeness through warmth (*β* = .11, *p* < .001, 95% CIs [.055, .168]) (i.e., expressed gratitude and apologies → warmth → closeness). Fourth, a message with gratitude and apologies was linked to closeness through conscientiousness (*β* = .04, *p* = .012, 95% CIs [.009, .076]) (i.e., expressed gratitude and apologies → conscientiousness → closeness). As indicated above, zero was not included in the 95% confidence intervals of all the significant indirect effects, so these effects were considered to be significant. As predicted in H2, warmth and conscientiousness mediated the link between different messages and closeness. However, agreeableness did not function as a mediator.

## DISCUSSION

Based on previous studies on the effects and usages of gratitude and apologies (Algoe et al., 2013; Lee et al., 2012), the current study hypothesized that expressed gratitude and apologies could increase a Japanese benefactor's perceived closeness toward a beneficiary. The predicted association between expressed gratitude/apologies and closeness was also hypothesized to be mediated by the benefactor's perception of the warmth, conscientiousness, and agreeableness of the beneficiary. Overall, the results indicated that expressed gratitude had more positive impacts on benefactors' perceptions of a beneficiary than expressed apologies. Specifically, benefactors who received a message showing gratitude and both gratitude and apologies reported higher levels of closeness toward a beneficiary compared with those who received a message with apologies and a message without either gratitude or apologies. Consistent with past findings (e.g., Algoe et al., 2013; Williams & Bartlett, 2015) and theoretical accounts (Thibaut & Kelley, 1959), this study revealed that expressed gratitude improved a benefactor's perception toward a beneficiary, which was represented as higher levels of closeness.

Contrary to the prediction, expressed apologies did not function to increase closeness. The results could be due to the insufficient positive outcome that a benefactor expects within a relationship with a beneficiary who apologizes. Consistent with core ideas from interdependence theory (Thibaut & Kelley, 1959), people are motivated to get closer to an individual when expected benefits are high and expected costs are low in a particular relationship with the individual (Aron et al., 2013). Thanking may help a benefactor realize the benefits in a relationship with a beneficiary because being thanked is associated with positive consequences for the benefactor, such as elevated levels of social worth and self‐efficacy (Grant & Gino, 2010). However, the main function of apologies is to help apologizers indicate that they take blame for negative outcomes (Chaudhry & Loewenstein, 2019), without offering positive consequences to the person being apologized to. Taken together, apologies may reduce a benefactor's perceived cost of the relationship associated with a beneficiary, but the communication may not help the benefactor forecast positive relational outcomes, unlike the case for thanking.

Another reason for the minimal effect of apologies might be that expressing apologies could indicate the psychological distance between an apology expressor and an apology receiver. Ide (1998) suggests that, in Japan, expressing apologies allows the expressor to place themselves in a humble position and make the utterance formal and polite. Therefore, in the current study, receiving apologies from a hypothetical friend might have made the participants perceive that the friend was too humble, formal, and polite. That perception may have made the participants experience psychological distance from the friend, which might be represented as suppressed perceptions of closeness. This study did not assess the psychological distance that apologies might imply, so future studies should measure the distance that different messages could indicate while taking into consideration the relationship between an apology expressor and an apology receiver.

Finally, expressed apologies could imply to the participants that the relationship was an exchange relationship rather than a communal relationship (Clark & Mills, 1979). In an exchange relationship, people assume that benefits are provided with the expectation of receiving a benefit in return and they are concerned with how much they receive in exchange for benefiting the other. On the other hand, in a communal relationship, people have a concern for the welfare of the other, so they have a positive attitude toward benefiting the other when there is a need for the benefit. In the current study, received apologies could make the participants believe that the relationship is an exchange relationship. That is, the participants may have felt that the hypothetical friend tried to take blame for negative outcomes (e.g., indebtedness) by apologizing. The interpretation could make the benefactor (participant) believe that the relationship functioned in a tit‐for‐tat fashion and find it difficult to facilitate closeness in the relationship. (If it is a communal relationship, a friend may not apologize because a benefactor would be happy to help the friend without thinking they are bothered.) In fact, expressed gratitude has been found to develop a communal relationship (Algoe, 2012; Lambert et al., 2010). In light of the above, expressed apologies might not generate enough impact to enhance closeness compared with gratitude because apologies may not be the best means to develop a communal relationship.

It should also be noted that apologies did not have an additional positive effect on closeness even when they were used with gratitude; that is, the results showed that there was not a significant difference between the score of closeness in the *gratitude* condition and that in the *both* condition. In Japan, it is not unnatural for people to use both gratitude and apologies to show gratitude (Lee et al., 2012; Yamamoto, 2003). Japanese people tend to be concerned about the indebtedness to others through communication (Benedict, 1946; Naito & Sakata, 2010), so they may use apologies to minimize the indebtedness while expressing gratitude. The results in this study suggest that such a strategy of using both gratitude and apologies did not generate additional positive impacts on closeness compared with the use of gratitude alone. Again, the reason may be that apologies theoretically may not function to help a benefactor expect positive outcomes within a relationship with an apologizer (Chaudhry & Loewenstein, 2019) even when apologies are used together with gratitude.

Further, the results of SEM indicated that the expression of gratitude and that of gratitude and apologies were linked to perceived closeness through warmth and conscientiousness. In other words, benefactors who received gratitude and both gratitude and apologies perceived higher levels of warmth and conscientiousness toward a beneficiary, and in turn those perceptions were associated with closeness. The results are generally consistent with the existing literature on expressed gratitude (e.g., Williams & Bartlett, 2015). What is not consistent with theoretical accounts is that perceived agreeableness did not function to mediate the link between expressed gratitude and closeness. The results indicate that scores of agreeableness in the *gratitude* and the *both* condition were higher than those in the *control* condition. However, the structural equation model depicted in Figure 1 shows an insignificant link between agreeableness and closeness. In other words, in this study thanking made a beneficiary appear agreeable, but that was not associated with closeness.

One of the possible explanations for the unobserved mediation role of agreeableness is that it might have lost predictive power because it was strongly correlated with warmth and conscientiousness. As shown in Table 3, agreeableness was significantly related to closeness, but it was not related to closeness when it was included in SEM with warmth and conscientiousness, as depicted in Figure 1. The literature indicates that agreeableness tends to be strongly correlated with warmth (Ames & Bianchi, 2008) and conscientiousness (Klimstra et al., 2013), so it makes sense that these two factors mostly explained the variation in closeness. The literature indicates that having a trait of agreeableness could help an individual appear to be a desirable relational partner (Jensen‐Campbell et al., 2010; Regan, 1998; Wortman & Wood, 2011), but some studies provide evidence showing that agreeable people may not bring about positive outcomes in a relationship (Bègue et al., 2015). Therefore, it is possible that in this study, participants unconsciously expected agreeable people who expressed gratitude not to bring about positive outcomes. However, these accounts are speculative, so future research should further investigate why agreeableness did not function as a mediator linking the association between expressed gratitude and closeness.

### Implications and limitations

One of the practical implications of these results is that if people receive a benefit, they should be encouraged to express gratitude, even in Japan, if they desire to develop a relationship with a benefactor. Apologies could be used to show gratitude, but they should be used together with thanking. The scores of closeness and conscientiousness in the *apology* condition were not significantly higher than those in the *control* condition. Even though the scores of warmth and agreeableness in the *apology* condition were significantly higher than those in the *control* condition, they were significantly lower than those in the *gratitude* condition and the *both* condition. That is, the positive effects of expressed apologies were limited compared with those of expressed gratitude.

Further, people should make sure that they appear warm and conscientious when they express gratitude with the purpose of forming a desirable relationship with a benefactor. The results did show that expressed gratitude made a benefactor feel close to the beneficiary through the elevated levels of perceived warmth and conscientiousness toward the beneficiary. In other words, even if people express gratitude to a benefactor, the development of the relationship between the beneficiary and the benefactor might not be expected if there is a failure to make the benefactor believe that the beneficiary is warm and conscientious. For instance, if a beneficiary expresses gratitude to a benefactor without smiling, which primarily functions to show the warmth of the expressor (Biancardi et al., 2017), the benefactor may not perceive the beneficiary to be warm, and that will undermine the positive effect of gratitude on the closeness perceived by the benefactor toward the beneficiary.

These contributions should be qualified in light of some limitations with this study. First, although this experimental research provided the most powerful design for testing the causal hypotheses that expressed gratitude leads to closeness, SEM did not directly test the causality among variables. Future studies should manipulate key mediators such as warmth and conscientiousness to investigate if they mediate the effect of expressed gratitude. Second, participants reported that the experimental situation was relatively realistic based on the results of items assessing realism, but the online survey this study employed might not have provided realistic interactions between a beneficiary and a benefactor. For example, participants received a reply from a hypothetical friend right after they sent a message with feedback on the statement of purpose, which would not happen in reality, because getting a response would be expected to take more time. Similar to in a past study (Lambert et al., 2010), future studies could ask dyads of friends to have a real interaction in which they use more gratitude and apologies depending on the experimental groups they are in, and see how the results could be consistent with what the current study observed. Third, the findings are based on a sample of Japanese individuals and may not be generalizable to people in other countries, such as Western countries, in which people are more likely to express gratitude rather than apologies (Lee et al., 2012). It would be ideal to recruit people from multiple countries and compare their perceptions in order to capture the full picture of culturally different effects of gratitude and apology in a benefit‐providing situation.

The final, critical factor that this study did not take into consideration was the relationship between a gratitude expressor and a gratitude receiver. This study set up a hypothetical situation in which participants interacted with a hypothetical friend. The participants in the study knew that the friend was in the same university, but the relationship between them and the friend was not clearly explained. However, the relationship could impact the results in many ways. For example, Japanese individuals are more inclined to apologize with an intention to thank superiors or seniors such as teachers (Iio, 2017). Therefore, if this study set up a situation in which participants communicated with a friend who was older than them, the effect of apologies could be intensified. Further, a study indicated that Chinese people experienced more *negative* feelings after they received gratitude from someone they felt close to than did Euro‐Canadians (Zhang et al., 2018). In other words, saying thank you to others might indicate the psychological distance between a gratitude expressor and a gratitude receiver in some cultures. Future studies should manipulate the relationship between a gratitude expressor and a gratitude receiver to fully capture the influences of expressed gratitude.

## CONCLUSION

The current study examined the effect of gratitude and apologies when a benefit is provided in Japan, specifically focusing on how these communications facilitate interpersonal relationships. The results showed that a message with gratitude and a message with gratitude and apologies had more positive effects on a benefactor's perception toward a beneficiary compared with a message with only apologies and a message without either gratitude or apologies. Further, expressed gratitude made a benefactor perceive a beneficiary to be warm and conscientious, and in turn these perceptions were linked to perceived closeness. This study hopefully paves the way for more research on how expressed gratitude and apologies facilitate or undermine interpersonal relationships.

## AUTHOR CONTRIBUTIONS

The author confirms sole responsibility for the following: study conception and design, data collection, analysis and interpretation of results, and manuscript preparation.

## FUNDING INFORMATION

Not applicable.

## CONFLICT OF INTEREST STATEMENT

The author declares that there is no conflict of interest.

## ETHICS STATEMENT

All procedures performed in studies involving human participants were in accordance with the ethical standards of the Institutional Research Committee of Nanzan University, to which the author belonged. The permission number is 20‐041.

## Data Availability

The data that support the findings of this study are openly available in The Open Science Framework (OSF) at https://mfr.osf.io/render?url=https://osf.io/yk8n9/?direct%26mode=render%26action=download%26mode=render.
